# GCK‐MODY in pregnancy: A pregnant woman with diabetes and a small‐for‐gestational‐age fetus

**DOI:** 10.1002/ccr3.6629

**Published:** 2022-12-05

**Authors:** Tiffany Tse Ling Yau, Stephanie Cheuk Yin Yu, Jenny Yeuk‐Ki Cheng, Jeffrey Sung Shing Kwok, Ronald Ching Wan Ma

**Affiliations:** ^1^ Department of Medicine and Therapeutics The Chinese University of Hong Kong, Prince of Wales Hospital Shatin Hong Kong; ^2^ Department of Chemical Pathology The Chinese University of Hong Kong, Prince of Wales Hospital Shatin Hong Kong; ^3^ Laboratory for Molecular Epidemiology in Diabetes, Li Ka Shing Institute of Health Sciences The Chinese University of Hong Kong Shatin Hong Kong; ^4^ Chinese University of Hong Kong‐Shanghai Jiao Tong University Joint Research Centre in Diabetes Genomics and Precision Medicine The Chinese University of Hong Kong Shatin Hong Kong

**Keywords:** fetal growth restriction, *GCK*‐MODY, insulin therapy, monogenic diabetes, pregnancy

## Abstract

Glucokinase‐maturity‐onset diabetes of the young (GCK‐MODY) is often misdiagnosed as other forms of diabetes. A 42‐year‐old pregnant lady with pre‐existing diabetes was treated with insulin during first trimester. Fetal growth restriction was noted since mid‐second trimester. Genetic testing suggested the diagnosis of GCK‐MODY.

## Graphical abstract

Glucokinase‐maturity‐onset diabetes of the young (GCK‐MODY) is under‐diagnosed. Patients with family history of young, non‐obese, and insulin‐independent diabetes should alert clinicians of the possibility of GCK‐MODY. It is important to diagnose timely, especially during pregnancy.

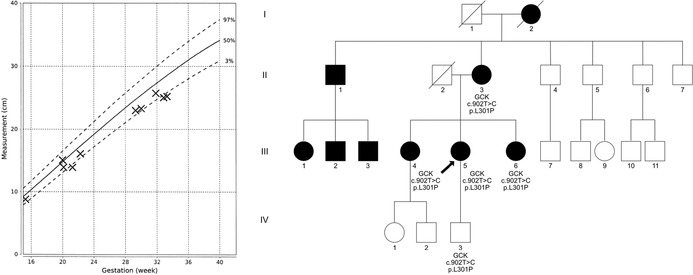

## 1 INTRODUCTION

Maturity‐onset diabetes of the young (MODY) is the most common form of monogenic diabetes mellitus. There are now 14 reported types of MODY.^1^ MODY caused by a heterozygous loss‐of‐function mutation of the *glucokinase* (*GCK*) gene (GCK‐MODY) has an estimated prevalence of 0.1% among the general population,^2^ accounting for 10% to 60% of MODY cases.^3^ Glucokinase is involved in the first step of glycolysis, which is the phosphorylation of glucose to glucose‐6‐phosphate. Patients with GCK‐MODY have mild stable fasting hyperglycemia, typically ranging from 5.5 to 8.0 mmol/L, which has been attributed to glucose regulation at a higher set‐point.^4^, ^5^ In general, pharmacological treatment is not required for GCK‐MODY because this group of patients seldom develops vascular complications.^5^ One exception is when a GCK‐MODY affected pregnant woman carries an unaffected fetus (i.e., fetus without maternal GCK mutation), when insulin treatment of maternal hyperglycemia is recommended.^3^ Making a diagnosis of GCK‐MODY is clinically challenging but important, particularly for pregnant women as this disease entity requires a different treatment approach compared with type 1, type 2, and gestational diabetes during pregnancy. However, it is not uncommon for GCK‐MODY patients to be misdiagnosed as type 1 or type 2 diabetes before pregnancy, or gestational diabetes during pregnancy.^6^

## 2 CASE PRESENTATION

A 42‐year‐old pregnant lady (gravida 1, para 0) visited the obstetrician for the management of diabetes in her first pregnancy. She was diagnosed with diabetes 1 year before getting pregnant and was managed as type 2 diabetes with metformin by a private medical practitioner. At that time, she presented with minor skin wounds. Yet, the concerned laboratory reports were not available to us. Her diabetic treatment was switched from metformin to insulin therapy when she was confirmed pregnant at 7th week of gestation. She was initially started on 6 units of bedtime Neutral Protamine Hagedorn (NPH) insulin and subsequently changed to 9 units of bedtime insulin glargine and 6 units of insulin lispro before lunch and dinner, respectively, since 10th week of gestation.

**FIGURE 1:**
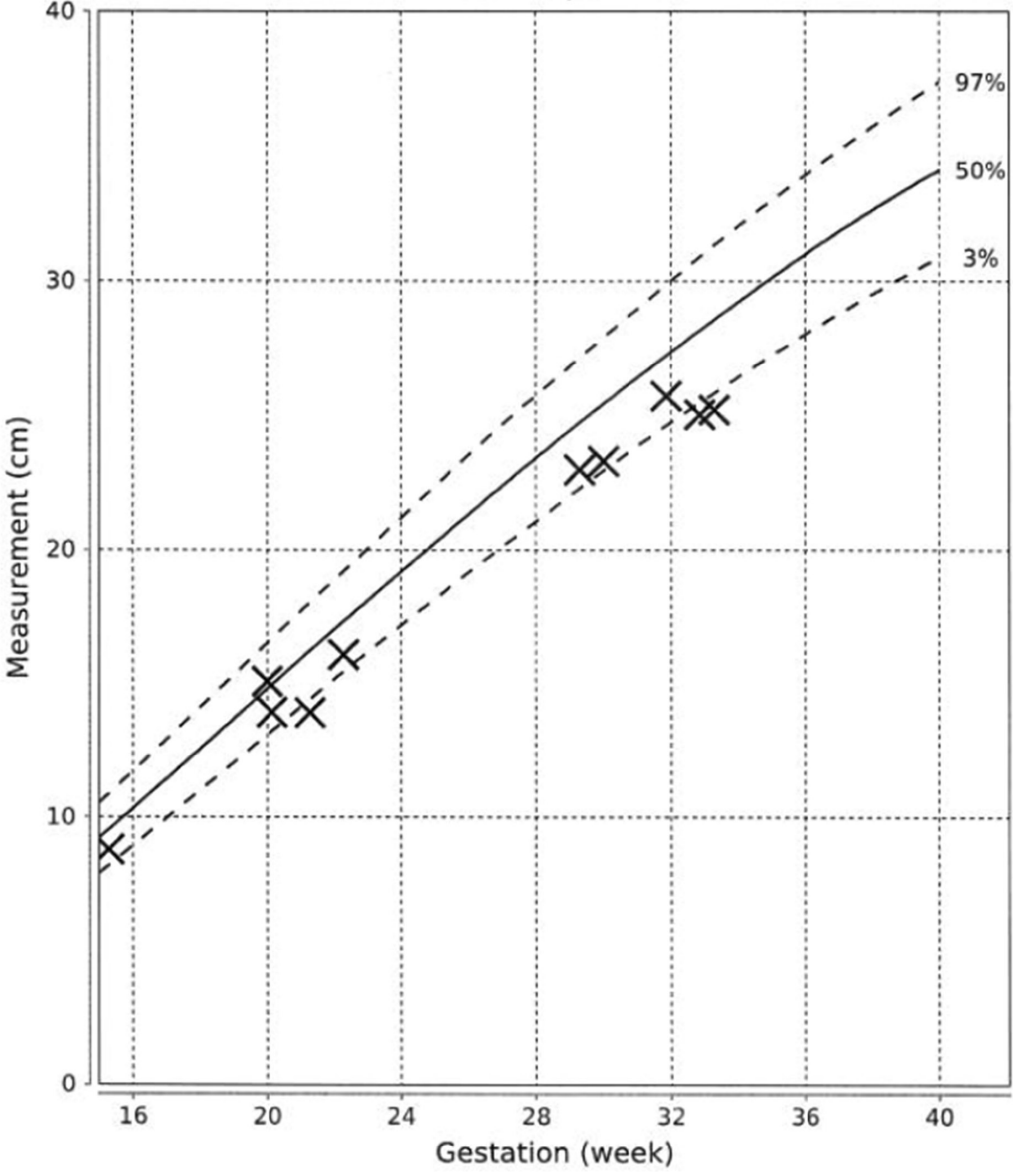
Measurements of abdominal circumference of the fetus in the index pregnancy (marked with crosses). The 3rd, 50th, and 97th centile lines were derived from data of a Chinese population.^20^

**FIGURE 2:**
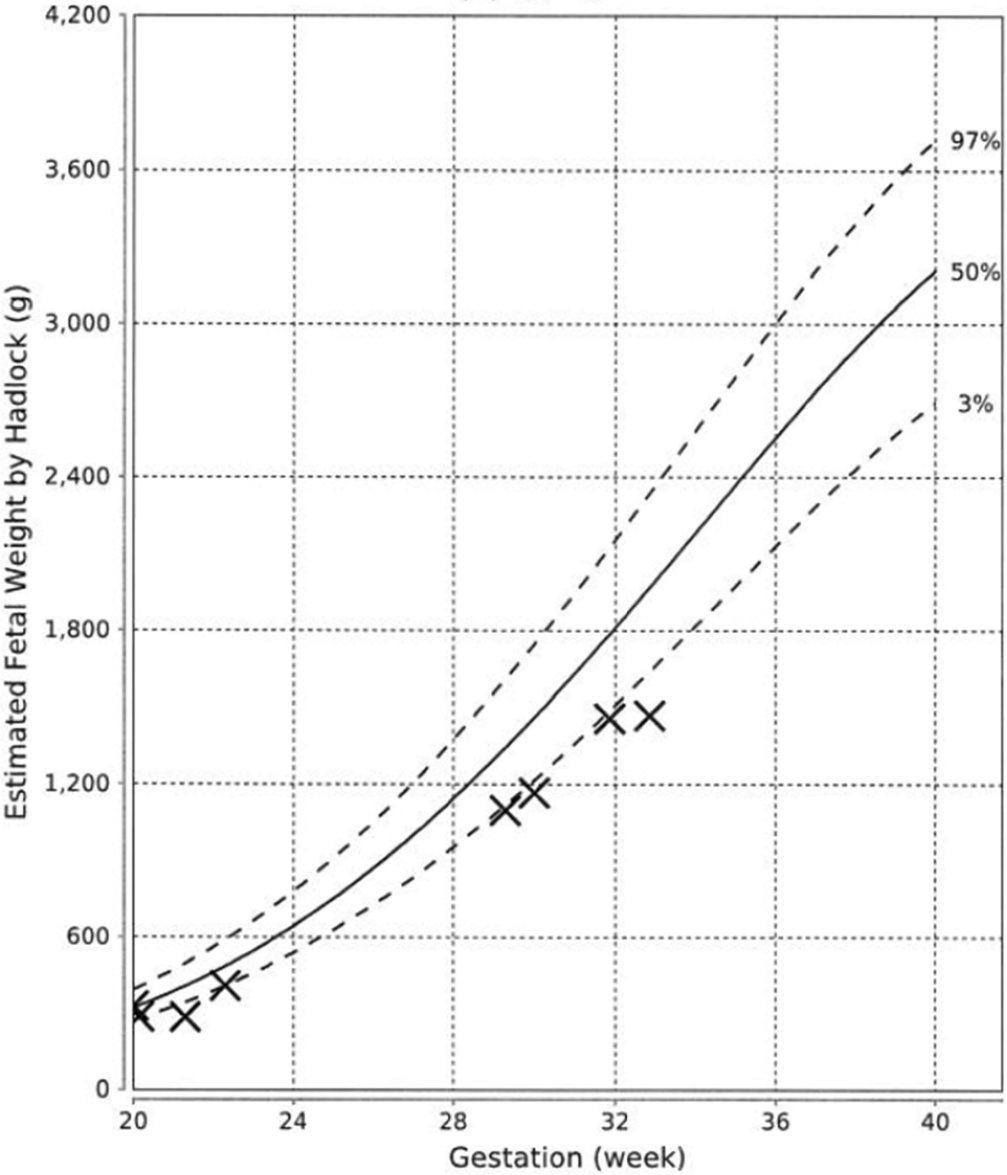
Estimated fetal weights (calculated by the Hadlock's formula) of the fetus in the index pregnancy (marked with crosses). The 3rd, 50th, and 97th centile lines were derived from data of a Chinese population.^21^

**FIGURE 3:**
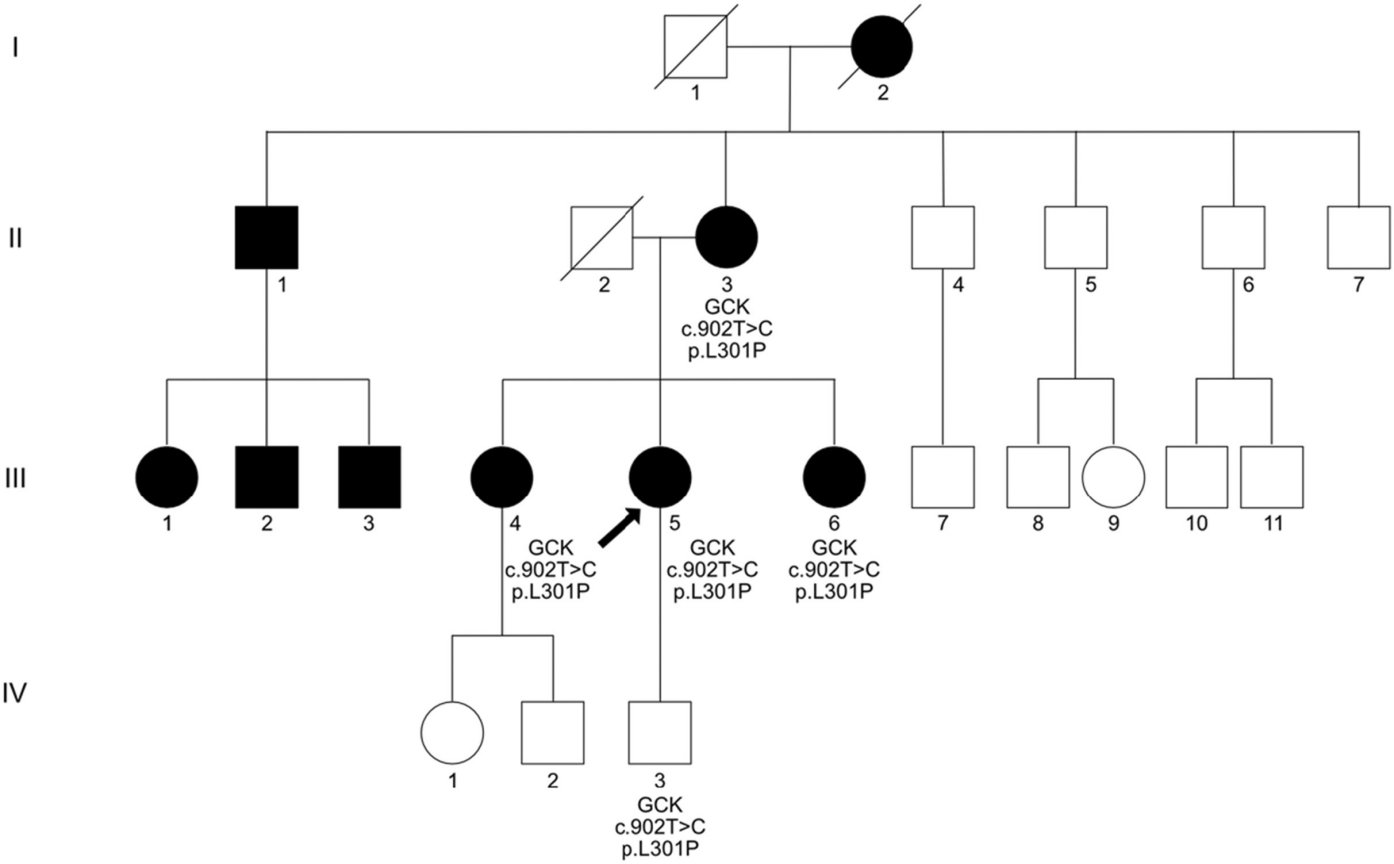
Pedigree showing the patient's family history of diabetes. Filled symbols indicate individuals with a known history of diabetes or impaired fasting glucose. Variants (if known) were marked below the symbols. I‐2: Maternal grandmother with diabetes and congestive heart failure; II‐1: Maternal uncle with diabetes diagnosed before 40 years old; II‐2: Proband's father died of intracerebral hemorrhage; II‐3: Proband's mother with diabetes diagnosed at 60 years old on metformin and pioglitazone; II‐4, 5, 6, 7: Maternal uncles whose medical history were unknown; III‐1, 2, 3: Maternal cousins with diabetes diagnosed before 40 years old; III‐4: Elder sister with impaired fasting glucose diagnosed at age 30 years old on diet control; III‐5: Proband; III‐6: Younger sister with impaired fasting glucose on diet control; III‐7, 8, 9, 10, 11: Maternal cousins whose medical history were unknown.

At 21st week of gestation, the abdominal circumference (AC) of the fetus was noted to be at the 10th percentile (Figure 1), with the estimated fetal weight (EFW) at the 3rd percentile (Figure 2). Further history taking revealed a strong maternal family history of diabetes (Figure 3). Of note, all family members with diabetes were non‐obese and had no complications of diabetes. On examination, the patient had a body mass index (BMI) of 22.6 kg/m^2^. She did not have any complications of diabetes.

## 3 INVESTIGATIONS

Laboratory evaluation at 7th week of gestation showed a hemoglobin A1c (HbA1c) of 6.5%. In view of the abnormal fetal growth parameters in this non‐obese mother with insulin‐treated diabetes who has a strong maternal family history of young uncomplicated diabetes, she was referred to an endocrinologist and the alternative diagnosis of GCK‐MODY was considered. After appropriate counseling, genetic testing was performed by Sanger sequencing of the promoter, all coding exons and splice sites of the *GCK* gene at 26th week of gestation, which revealed a heterozygous novel missense variant NM_000162.5:c.902T>C, p.(Leu301Pro). This variant was classified as likely pathogenic as per guidelines from the American College of Medical Genetics and Genomics and the Association for Molecular Pathology (ACMG/AMP).^7^

## 4 TREATMENT

In view of the compatible clinical picture together with the likely pathogenic variant identified, the patient was managed as GCK‐MODY. The fetus was suspected to be an affected carrier given fetal growth restriction; thus insulin treatment was stopped from 29th week of gestation onwards. Home capillary blood glucose (CBG) monitoring showed similar levels of fasting (5.3–7.4 mmol/L vs. 5.2–6.8 mmol/L) and 2 h post‐prandial glucose (6.0–11.0 mmol/L vs. 6.8–11.0 mmol/L) before and after withdrawal of insulin therapy. A male baby was delivered via an emergency caesarean section at 33rd week of gestation due to preterm premature rupture of membrane. The birth weight was 1.815 kg (below the 3rd percentile).

## 5 OUTCOME AND FOLLOW‐UP

After delivery, the patient was not on any treatment for glycaemic control. A 75 g oral glucose tolerance test (OGTT) at 5 weeks postpartum showed a fasting plasma glucose of 6.8 mmol/L and a 2‐h plasma glucose of 11.9 mmol/L. The HbA1c was 6.9%. Blood samples from the patient's mother, elder sister, younger sister, and the newborn proceeded with biochemical and genetic analyses. The patient's mother was on metformin and pioglitazone at the time of biochemical analysis, while the patient and her two sisters were not on any anti‐diabetic treatment. The patient's mother and two sisters had impaired fasting glucose levels of 6.4 to 6.5 mmol/L. Her elder sister showed a 2‐h plasma glucose of 13.0 mmol/L during the OGTT while her mother and younger sister showed a 2‐h plasma glucose of 8.4 and 8.5 mmol/L, respectively. The HbA1c values were 6.6%, 6.1%, and 6.0% for her mother, elder sister, and younger sister, respectively. The same variant *GCK* c.902T > C, p.(Leu301Pro) was identified in the patient's mother, elder sister, younger sister and the newborn, suggesting co‐segregation of the variant with the phenotype in this family. The latest fasting blood glucose and HbA1c of the patient at 1 year after delivery were 7.0 mmol/L and 6.6%, respectively.

## 6 DISCUSSION

In this report, we presented a case of GCK‐MODY who was initially treated as type 2 diabetes with insulin in pregnancy until a diagnosis of GCK‐MODY was made in the early third trimester. The clinical picture of fetal growth restriction in an insulin‐treated diabetic pregnant woman with a strong maternal family history of young, non‐obese and uncomplicated diabetes prompted the endocrinologists to consider GCK‐MODY in this patient and her family.

This case report has reiterated two important clinical challenges in the management of GCK‐MODY in pregnancy. Firstly, many cases of GCK‐MODY are unrecognized or misdiagnosed before and during early pregnancy. Secondly, the fetal GCK genotype is usually not known in utero, which may adversely affect the fetal growth for cases with mothers suffering from GCK‐MODY.

GCK‐MODY is the second most common MODY subtype in Chinese population.^5^ Clinical features of GCK‐MODY include a young age at diagnosis, being non‐obese, having persistent non‐progressive hyperglycemia, and absence of complications of diabetes. Biochemical features include a mild fasting hyperglycemia in the range of 5.5–8.0 mmol/L, an increment in plasma glucose of less than 4.6 mmol/L in an OGTT, and a HbA1c value in the range of 5.6%–7.6%.^5^ However, patients could have variable phenotypes.^8^ For our patient, the increment in plasma glucose after OGTT was 5.1 mmol/L. This relatively large increase in plasma glucose after OGTT might be explained by the impairment of hepatic glucokinase as observed in some GCK‐MODY patients, which leads to impaired glycogenesis and increased gluconeogenesis after meals.^8^ Family history of diabetes or impaired fasting glucose is also of paramount importance, apart from clinical history and biochemical investigation, as strong family history of young diabetes might suggest the possibility of monogenic diabetes, just like our case.

With reference to the Human Gene Mutation Database,^9^ 1001 mutations have been identified in the GCK gene, with 711 being missense or nonsense mutations. The variant NM_000162.5:c.902T>C, p.(Leu301Pro) is absent in multiple genetic databases and has not been reported in the literature. It is located in a mutational hot spot where no benign variants have been reported. In silico analysis based on multiple computational tools also suggests the pathogenicity of this variant. Meanwhile, most of the missense variants in the GCK gene are pathogenic in nature. With the above evidence of pathogenicity, this variant is classified as likely pathogenic according to ACMG/AMP guideline.^7^

Around 1%–2% of patients with gestational diabetes have GCK‐MODY.^3^ Due to the possible overlapping clinical features, misdiagnosis of GCK‐MODY as other forms of diabetes is not uncommon.^6^ Clinical practice guidelines for selecting patients for genetic testing of GCK‐MODY have existed for a decade.^2^, ^10^ Ellard et al.^10^ suggested testing for GCK mutations in pregnant women who have persistent elevations of fasting glucose ranging from 5.5 to 8 mmol/L, an increment in plasma glucose of less than 4.6 mmol/L in OGTT, and a positive family history. More recently, Chakera et al. proposed combined criteria of fasting glucose of greater than 5.5 mmol/L and a pre‐pregnancy BMI of less than 25 kg/m^2^ for the screening of GCK‐MODY in pregnancy.^2^ Such combined criteria achieved a sensitivity of 68% and a specificity of 96%, with a number needed to test of 2.7 women with gestational diabetes to identify one case of GCK‐MODY. However, since the study by Chakera et al. was conducted in a Caucasian population, further studies would be required to determine whether the same screening criteria is applicable to other ethnic populations. To further enhance the identification of patients for genetic testing of GCK‐MODY, a thorough understanding of the clinical and biochemical characteristics of GCK‐MODY, a heightened awareness and suspicion by clinicians in general practice, general medicine, endocrinology, and obstetrics would be beneficial.

In pregnancies of GCK‐MODY mothers, fetal growth is largely dependent on the fetal GCK genotype.^5^ Endogenous insulin from fetal pancreas has a stimulatory effect on fetal growth.^11^ A normal level of fetal insulin secretion is required for a normal fetal growth. When a GCK‐MODY affected pregnant woman carries an unaffected fetus, the mild maternal hyperglycemia will cause increased insulin secretion from the fetus, resulting in an excessive insulin‐stimulated fetal growth.^12^ In contrast, when a GCK‐MODY affected pregnant woman carries a GCK‐MODY affected fetus, the fetal insulin secretion and hence, fetal growth will be normal if the maternal hyperglycemia is not treated since the fetus has the same elevated glucose set‐point as the mother.^13^, ^14^, ^15^

When GCK‐MODY affected mothers were inadvertently treated with insulin during pregnancy, it might result in fetal growth restriction if fetuses also carry the GCK mutation.^5^, ^13^ In one study, among the 28 GCK‐MODY affected mothers with GCK‐MODY affected fetus, those fetuses whose mother were treated with insulin during pregnancy have lower birthweights compared with those fetuses whose mothers received no treatment during pregnancy.^16^ As subsequently revealed by genetic testing after birth in this case, the newborn has inherited the same GCK variant from his mother (the index patient), likely explaining the fetal growth restriction as the mother was treated with insulin.

The current clinical practice for the management of GCK‐MODY pregnancy is to predict the fetal GCK genotype based on the fetal phenotype in utero. Any fetus with an AC greater than the 75th percentile on ultrasound during the second trimester would be inferred not to have inherited the GCK gene mutation from mother. In such cases, the mother may be treated with insulin to control maternal glycaemia.^3^, ^17^ However, it has been reported that high doses of insulin are often required to overcome the elevated glucose set point in patients with GCK‐MODY, which resulted in frequent episodes of hypoglycemia.^14^, ^18^ Our patient did not experience any hypoglycemia symptoms while she was on insulin therapy during pregnancy because she had been on a relatively low dose of insulin (the maximum dose received was 9 units of basal insulin and 6 units of mealtime insulin) all along. Despite a suboptimal glycemic control was noted at 24 weeks of gestation, the insulin therapy was not escalated because the endocrinologist already suspected a diagnosis of GCK‐MODY for this patient. Another management strategy to consider is to induce labor at 38 weeks to reduce the risk of birth trauma associated with fetal macrosomia.^3^, ^17^ Recently, Caswell et al.^19^ reported a highly sensitivity and accurate droplet digital PCR‐based assay for cell‐free DNA analysis in maternal plasma for the non‐invasive prenatal determination of fetal GCK genotype in GCK‐MODY affected pregnant women. Yet, the performance and the actual diagnostic impact of such approach would require validation by studies with larger sample cohorts.

## AUTHOR CONTRIBUTIONS

All authors were involved in the conceptualization, acquisition, and analysis or interpretation of data. TTL Yau, SCY Yu, and JYK Cheng drafted the manuscript. JSS Kwok and RCW Ma reviewed the manuscript.

## CONFLICT OF INTEREST

There are no conflicts of interest to be declared.

## CONSENT

Written informed consent was obtained from the patient to publish this report in accordance with the journal's patient consent policy.

## Data Availability

Data available on request due to privacy/ethical restrictions.
